# Knockdown of AMPKα2 Promotes Pulmonary Arterial Smooth Muscle Cells Proliferation via mTOR/Skp2/p27^Kip1^ Signaling Pathway

**DOI:** 10.3390/ijms17060844

**Published:** 2016-05-31

**Authors:** Rui Ke, Lu Liu, Yanting Zhu, Shaojun Li, Xinming Xie, Fangwei Li, Yang Song, Lan Yang, Li Gao, Manxiang Li

**Affiliations:** 1 Department of Respiratory and Critical Care Medicine, The First Affiliated Hospital of Xi’an Jiaotong University, Xi’an 710061, China; ke19870912@163.com (R.K.); liulu290@126.com (L.L.); zhuyanting0816@163.com (Y.Z.); wdm19870914@163.com (S.L.); xiexinming1986@163.com (X.X.); lifangwei80@163.com (F.L.); wyshzl@163.com (Y.S.); yanglanmed@163.com (L.Y.); 2 Division of Allergy and Clinical Immunology, Department of Medicine, The Johns Hopkins University School of Medicine, Baltimore, MD 21224, USA; lgao2@jhmi.edu

**Keywords:** AMPK, pulmonary arterial smooth muscle cells, proliferation, mTOR, Skp2

## Abstract

It has been shown that activation of adenosine monophosphate-activated protein kinase (AMPK) suppresses proliferation of a variety of tumor cells as well as nonmalignant cells. In this study, we used post-transcriptional gene silencing with small interfering RNA (siRNA) to specifically examine the effect of AMPK on pulmonary arterial smooth muscle cells (PASMCs) proliferation and to further elucidate its underlying molecular mechanisms. Our results showed that knockdown of AMPKα2 promoted primary cultured PASMCs proliferation; this was accompanied with the elevation of phosphorylation of mammalian target of rapamycin (mTOR) and S-phase kinase-associated protein 2 (Skp2) protein level and reduction of p27^Kip1^. Importantly, prior silencing of mTOR with siRNA abolished AMPKα2 knockdown-induced Skp2 upregulation, p27^Kip1^ reduction as well as PASMCs proliferation. Furthermore, pre-depletion of Skp2 by siRNA also eliminated p27^Kip1^ downregulation and PASMCs proliferation caused by AMPKα2 knockdown. Taken together, our study indicates that AMPKα2 isoform plays an important role in regulation of PASMCs proliferation by modulating mTOR/Skp2/p27^Kip1^ axis, and suggests that activation of AMPKα2 might have potential value in the prevention and treatment of pulmonary arterial hypertension.

## 1. Introduction

Pulmonary arterial hypertension (PAH) is a common clinical syndrome characterized by sustained elevation of pulmonary vascular resistance and increased pulmonary arterial pressure, leading to right heart failure and ultimately death [1,2]. Pulmonary vascular remodeling characterized by thickening of all layers of vascular wall is a hallmark of all types of PAH [3]. Pulmonary arterial smooth muscle cells (PASMCs) proliferation *in situ* and migration to intima is critical to the pathogenesis of pulmonary vascular remodeling [4]. The deregulation of multiple signaling pathways in PASMCs is convinced to be associated with excessive proliferation of PASMCs [5,6]. Therefore, strategies either to enhance the function of anti-proliferative cascades or to inhibit pro-proliferative signaling pathways are important in the amelioration of pulmonary vascular remodeling and therefore the management of PAH.

Adenosine monophosphate-activated protein kinase (AMPK) is a key metabolic and redox sensor that has emerged as a critical regulator of cell proliferation and apoptosis [7]. AMPK exists as a heterotrimeric complex with a catalytic subunit α and two regulatory subunits β and γ [8]. Pathological changes associated with increased AMP to ATP ratio, such as nutrient deprivation, hypoxia, ischemia, heat shock and oxidative stress, cause AMPK activation [8,9]. AMPK is also found to be activated by several chemical compounds [10,11] and signaling pathways [12] independent of energy imbalance. Activation of AMPK regulates diverse cellular processes including cell migration, proliferation, apoptosis and survival [13]. Studies have shown that AMPK activation suppresses proliferation of a variety of tumor cells as well as nonmalignant cells, which indicates that activation of AMPK is a promising strategy for the treatment of cancer as well as other human diseases [14,15,16].

Recent studies have employed AMPK activators such as 5-aminoimidazole-4-carboxamide ribonucleoside (AICAR) and metformin to demonstrate that activation of AMPK suppresses PASMCs proliferation [17,18]. Studies in animal model have also found that activation of AMPK by metformin inhibits the development of PAH by suppressing pulmonary vascular remodeling [19]. However, downstream targets regulated by AMPK to confer its roles in PAH are not fully clarified yet; in addition, pharmacological modulation of AMPK is not entirely specific for AMPK and has off-target effects [20,21]. RNA interference (RNAi) is a simple and effective method for silencing gene expression and has great advantages over traditional approaches for examining the role of a specific molecule in the development of a particular disease [22]. In the present study, we used post-transcriptional gene silencing with small interfering RNA (siRNA) to selectively inhibit AMPK in primary cultured PASMCs, and specifically examined the effect of AMPK on PASMCs proliferation, and the molecular mechanisms underlying this effect were further explored.

## 2. Results

### 2.1. Knockdown of Adenosine Monophosphate-Activated Protein Kinase α2 (AMPKα2) Promotes Pulmonary Arterial Smooth Muscle Cells (PASMCs) Proliferation

**Figure 1:**
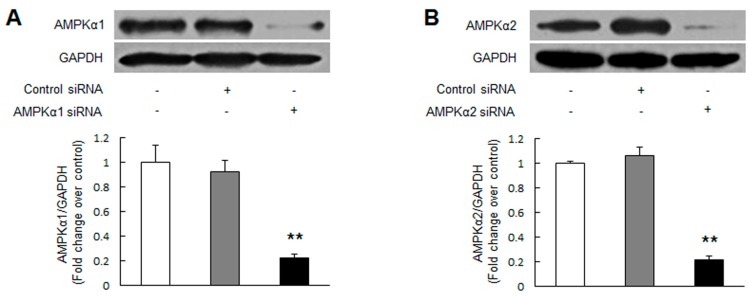

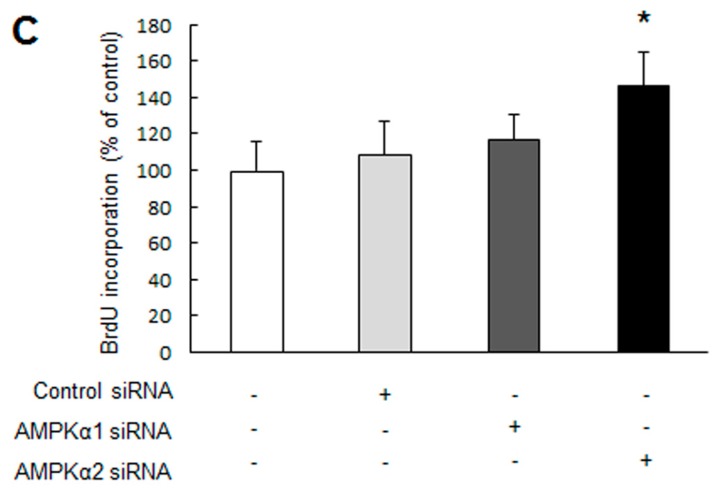
Knockdown of adenosine monophosphate-activated protein kinase α2 (AMPKα2) promotes pulmonary arterial smooth muscle cells (PASMCs) proliferation. Primary cultured PASMCs were transfected with AMPKα1 or AMPKα2 sequence-specific small interfering RNA (siRNA) and non-targeting siRNA. Protein levels of AMPKα1 (**A**) and AMPKα2 (**B**) were determined by immunoblotting (*n* = 3 in each group); (**C**) PASMCs were transfected with indicated siRNA, cells proliferation was examined by BrdU incorporation assay (*n* = 4 in each group). +, with indicated siRNA transfection; −, without siRNA transfection; * *p* < 0.05 *versus* control; ** *p* < 0.01 *versus* control.

To specifically examine the effect of AMPK on PASMCs proliferation, siRNA-mediated knockdown of either AMPKα1 or AMPKα2 was performed. As depicted in Figure 1A,B, transfection of the corresponding specific siRNA reduced the levels of AMPKα1 and AMPKα2 protein by 78% and 79%, respectively (*p* < 0.01 *versus* control). Non-targeting siRNA transfection did not reduce AMPKα1 and AMPKα2 protein expression. Figure 1C indicates that PASMCs lacking AMPKα2 protein exhibited an elevated cells proliferation assessed by BrdU incorppration assay, which was 1.46-fold increase over control (*p* < 0.05), while transfection of PASMCs with AMPKα1 siRNA or non-targeting siRNA did not affect cells proliferation. These results suggest that knockdown of AMPKα2 induces PASMCs proliferation.

### 2.2. Knockdown of AMPKα2 Increases the Phosphorylation of Mammalian Target of Rapamycin (mTOR), Upregulates S-Phase Kinase-Associated Protein 2 (Skp2) and Downregulates p27^Kip1^

**Figure 2:**
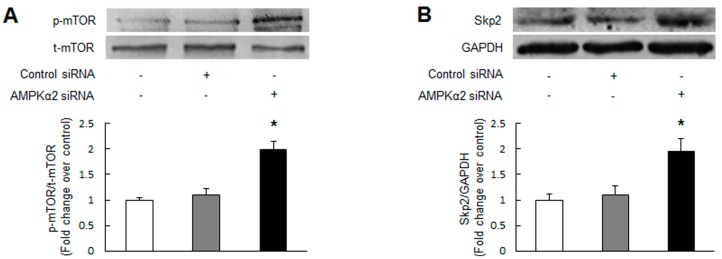

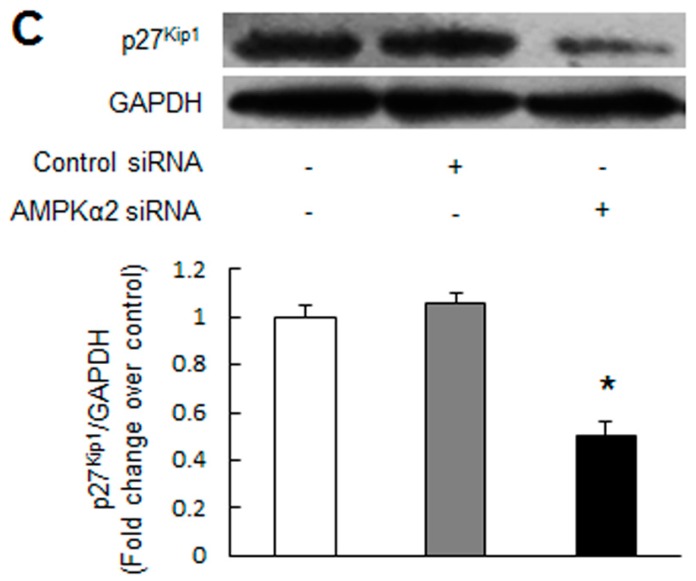
Knockdown of AMPKα2 increases the phosphorylation of mammalian target of rapamycin (mTOR), upregulates S-phase kinase-associated protein 2 (Skp2) and downregulates p27^Kip1^. Primary cultured PASMCs were transfected with AMPKα2 specific siRNA and non-targeting siRNA. Phosphorylation of mTOR (**A**) and protein levels of Skp2 (**B**) and p27^Kip1^ (**C**) were determined by immunoblotting (*n =* 3 in each group). +, with indicated siRNA transfection; −, without siRNA transfection; * *p* < 0.01 *versus* control.

Mammalian target of rapamycin (mTOR) is a serine/threonine kinase that positively regulates cell growth, proliferation, and survival [23]. Several groups have reported that activation of AMPK targets on mTOR to suppress its function and further inhibits a variety of cells proliferation [24,25,26]. To investigate whether knockdown of AMPK causes activation of mTOR in PASMCs, AMPKα2 was silenced by siRNA transfection and mTOR activity was measured using immunoblotting. Figure 2A indicates that the phosphorylation of mTOR, which is an indicator of mTOR intrinsic catalytic activity, was elevated in AMPKα2 knockdown PASMCs with a 1.99-fold increase compared with control (*p* < 0.01), suggesting that knockdown of AMPKα2 causes unrestrained mTOR activation in PASMCs.

p27^Kip1^ is one of the cyclin-dependent kinases (CDKs) inhibitors that plays a critical role in regulating cell cycle progression in mammalian cells [27]. It has been shown that inhibition of S-phase kinase-associated protein 2 (Skp2)-mediated p27^Kip1^ degradation underlies AMPK suppression of several types of cells proliferation [17,28,29]. Here, we examined whether the changes of Skp2 and p27^Kip1^ are also associate with the effect of AMPKα2 knockdown on PASMCs proliferation. As shown in Figure 2B, cells lacking AMPKα2 by siRNA transfection demonstrated a 1.95-fold increase in Skp2 protein expression (*p* < 0.01 *versus* control). Non-targeting siRNA transfection did not affect Skp2 protein level. Figure 2C indicates that protein level of p27^Kip1^ was reduced in AMPKα2 knockdown PASMCs, which was 0.50-fold decrease compared with control (*p* < 0.01), while non-targeting siRNA transfection did not change p27^Kip1^ expression. These findings suggest that knockdown of AMPKα2 upregulates Skp2 and downregulates p27^Kip1^ in PASMCs.

### 2.3. mTOR Mediates AMPKα2 Knockdown-Induced Skp2 Upregulation, p27^Kip1^ Downregulation and PASMCs Proliferation

**Figure 3:**
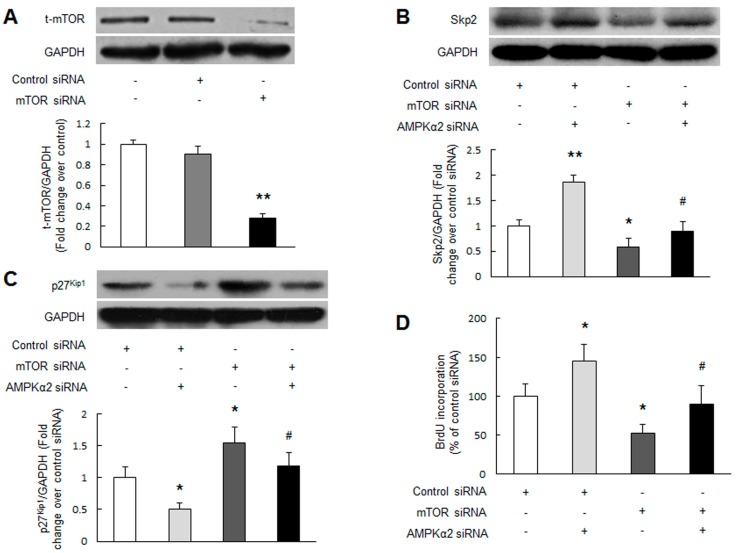
mTOR mediates AMPKα2 knockdown-induced Skp2 upregulation, p27^Kip1^ downregulation and PASMCs proliferation. (**A**) Primary cultured PASMCs were transfected with mTOR sequence-specific siRNA and non-targeting siRNA. mTOR protein level was measured using immunoblotting (*n* = 3 in each group); (**B**–**D**) PASMCs were transfected with non-targeting siRNA or mTOR specific siRNA, and followed by transfection with or without AMPKα2 siRNA. Protein level of Skp2 (**B**) and p27^Kip1^ (**C**) were determined using immunoblotting (*n* = 3 in each group). Cell proliferation was examined by BrdU incorporation assay (**D**) (*n* = 4 in each group). +, with indicated siRNA transfection; −, without siRNA transfection; * *p* < 0.05 *versus* control siRNA transfection; ** *p* < 0.01 *versus* control siRNA transfection; # *p* < 0.01 *versus* control siRNA + AMPKα2 siRNA transfection.

Studies have shown that mTOR plays an important role in regulating Skp2 and p27^Kip1^ in malignant cells [30,31,32]. To determine whether mTOR mediates the effect of AMPKα2 knockdown on Spk2 and p27^Kip1^ modulation in PASMCs, silencing of mTOR was applied. Figure 3A indicates that mTOR specific siRNA transfection reduced mTOR protein level to 28 percent of control (*p* < 0.01), while non-targeting siRNA did not affect mTOR protein expression. Figure 3B,C show that AMPKα2 knockdown notably upregulated Skp2 expression (*p* < 0.01 *versus* control siRNA transfection) and reduced p27^Kip1^ protein level (*p* < 0.05 *versus* control siRNA transfection), while pre-silencing of mTOR decreased Skp2 expression from 1.87-fold to 0.89-fold over control siRNA and increased p27^Kip1^ level from 0.50-fold to 1.19-fold over control siRNA, respectively (*p* < 0.01 *versus* control siRNA + AMPKα2 siRNA transfection). We also observed that silencing of mTOR alone reduced Skp2 and increased p27^Kip1^ protein level (*p* < 0.05 *versus* control siRNA transfection), suggesting that there is a basal function of mTOR in PASMCs. These results suggest that activation of mTOR is one of major protein responsible for AMPKα2 knockdown-induced Skp2 upregulation and p27^Kip1^ downregulation.

To further confirm whether activation of mTOR mediates AMPKα2 knockdown-induced PASMCs proliferation, BrdU incorporation rate was determined in PASMCs transfected with non-targeting siRNA or mTOR siRNA followed with or without AMPKα2 knockdown. Figure 3D shows that AMPKα2 knockdown triggered a 1.45-fold increase in cells proliferation (*p* < 0.05 *versus* control siRNA transfection), while pre-silencing of mTOR reduced PASMCs proliferation to 0.90-fold over control siRNA (*p* < 0.01 *versus* control siRNA + AMPKα2 siRNA transfection). Silencing of mTOR alone reduced basal PASMCs proliferation (*p* < 0.05 *versus* control siRNA transfection). Together, these observations indicate that mTOR mediates AMPKα2 knockdown-induced PASMC proliferation by regulating Skp2 and p27^Kip1^.

### 2.4. Skp2 Mediates AMPKα2 Knockdown-Induced p27^Kip1^ Reduction and PASMCs Proliferation

**Figure 4:**
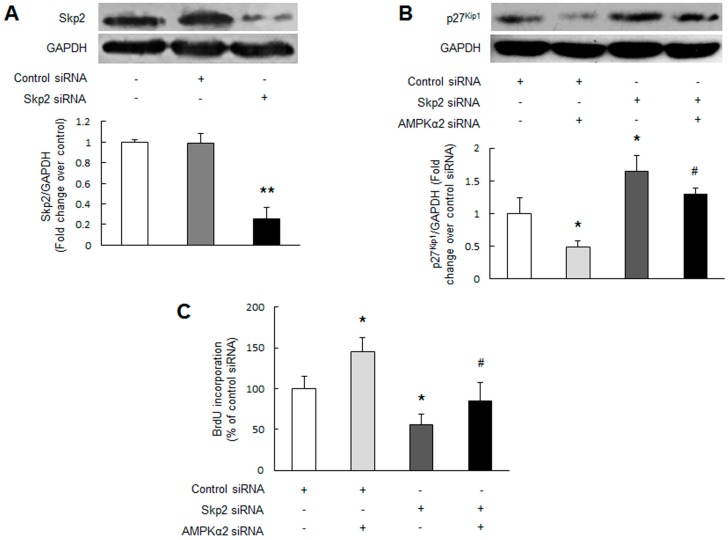
Skp2 mediates AMPKα2 knockdown-induced p27^Kip1^ reduction and PASMCs proliferation. (**A**) Primary cultured PASMCs were transfected with Skp2 sequence-specific siRNA and non-targeting siRNA, the silencing efficiency was assessed by immunoblotting (*n* = 3 in each group); (**B**,**C**) PASMCs were transfected with non-targeting siRNA or Skp2 specific siRNA, and followed with or without AMPKα2 siRNA transfection. Protein level of p27^Kip1^ was examined using immunoblotting (**B**) (*n* = 3 in each group). Cell proliferation was measured by BrdU incorporation assay (**C**) (*n* = 4 in each group). +, with indicated siRNA transfection; −, without siRNA transfection; * *p* < 0.05 *versus* control siRNA transfection; ** *p* < 0.01 *versus* control siRNA transfection; # *p* < 0.01 *versus* control siRNA + AMPKα2 siRNA transfection.

Skp2 is the main rate-limiting regulator of p27^Kip1^ ubiquitylation and degradation in various cell types [28,29]. To examine whether Skp2 mediates AMPKα2 knockdown-induced p27^Kip1^ reduction, depletion of Skp2 was applied. Figure 4A indicates that Skp2 specific siRNA transfection reduced Skp2 protein level to 25 percent of control (*p* < 0.01), while non-targeting siRNA did not affect Skp2 protein level. Figure 4B shows that AMPKα2 knockdown significantly reduced p27^Kip1^ protein level (*p* < 0.05 *versus* control siRNA transfection), while prior depletion of Skp2 abrogated the reduction of p27^Kip1^, which increased from 0.50-fold to 1.30-fold over control siRNA (*p* < 0.01 *versus* control siRNA + AMPKα2 siRNA transfection). Loss of basal Skp2 alone also increased p27^Kip1^ protein level (*p* < 0.05 *versus* control siRNA transfection). These results suggest that Skp2 specifically mediates AMPKα2 knockdown-induced p27^Kip1^ reduction.

To further verify the involvement of Skp2 in AMPKα2 knockdown-induced PASMCs proliferation, PASMCs were transfected with non-targeting siRNA or Skp2 siRNA followed with or without AMPKα2 knockdown. Figure 4C demonstrated that AMPKα2 knockdown markedly stimulated PASMCs proliferation (*p* < 0.05 *versus* control siRNA transfection), while prior depletion of Skp2 inhibited PASMCs proliferation induced by AMPKα2 knockdown, which declined from 1.45-fold to 0.85-fold over control siRNA (*p* < 0.01 *versus* control siRNA + AMPKα2 siRNA transfection). Lacking Skp2 protein alone also reduced basal PASMCs proliferation (*p* < 0.05 *versus* control siRNA transfection). Collectively, these results suggest that Skp2-induced p27^Kip1^ reduction mediates AMPKα2 knockdown-stimulated PASMCs proliferation.

## 3. Discussion

In the present study, we have provided direct evidence that knockdown of AMPKα2 by siRNA stimulates primary cultured PASMCs proliferation; this effect is coupled to the particular activation of mTOR and subsequent upregulation of Skp2 and reduction of p27^Kip1^. The study suggests that activation of AMPKα2 might have potential value in the management of PAH by modulation of pulmonary vascular remodeling.

AMPK is a crucial energy sensor that regulates cellular and whole-body energy balance [8]. Although best known for its effects on metabolism, AMPK has been recently found to have many other functions such as regulation of cell proliferation, apoptosis and migration [13]. Emerging studies have shown that activation of AMPK benefits a variety of cancer as well as other diseases by suppressing proliferation of tumor cells as well as nonmalignant cells [14,15,16]. In this study, we used post-transcriptional gene silencing with siRNA to specifically examine the effect of AMPK on PASMCs proliferation. Our results demonstrated that knockdown of AMPKα2 isoform promotes PASMCs proliferation, indicating the anti-proliferative action of AMPKα2 in PASMCs. AMPK has numerous targets in pathways involved in growth regulation [33]. mTOR, a key positive regulator of cell growth, proliferation, and survival, has been proved to be one of the most characterized downstream effectors of AMPK [24,25,26]. mTOR controls proteins synthesis required for cell proliferation and is implicated in a variety of human disorders, including cancer, cardiovascular disease, autoimmunity, metabolic disorders and inherited disease [23,34,35]. Recent studies have also found that mTOR activation promotes PASMCs proliferation and is associated with pulmonary vascular remodeling in PAH [36,37]. mTOR is negatively regulated by tuberous sclerosis complex 1 (TSC1)/TSC2 [38]. In the majority of cells under stress conditions, activation of AMPK phosphorylates TSC2 leading to TSC1/TSC2-dependent suppression of mTOR to inhibit cell proliferation [38,39]. The current study indicated that knockdown of AMPKα2 caused unrestrained mTOR activation and increased PASMCs proliferation. Loss of mTOR with siRNA abolished AMPKα2 knockdown-induced PASMCs proliferation. This indicates that mTOR might be a direct target of AMPKα2 in regulation of PASMCs proliferation. 

Mammalian cell proliferation is delicately regulated by cell-cycle regulatory proteins including CDKs and CDK inhibitors [40]. Skp2, a F-box component of Skp1/Cullin/F-box protein (SCF)-type ubiquitin ligase, is responsible for the ubiquitination and proteasomal degradation of cell-cycle regulators such as the CDK inhibitor p27^Kip1^ [41]. Upregulation of Skp2 has been observed in various malignant tumors [42,43]. Moreover, tumors overexpressing Skp2 are strongly associated with low p27^Kip1^ levels and poor disease-free and overall survival [44]. Inhibition of Skp2 prevents p27^Kip1^ degradation and suppresses proliferation of many cell types [45,46]. p27^Kip1^ as a CDK inhibitor binds to and prevents the activation of cyclin E-CDK2 and cyclin D-CDK4 complexes, therefore blocks the G1-S transition of cell cycle and suppresses cell proliferation [47,48]. Our study showed that PASMCs lacking AMPKα2 exhibited a significant elevation of Skp2 protein level; this was accompanied with reduction of p27^Kip1^ and proliferation of PASMCs. Silencing of mTOR attenuated the changes of Skp2 and p27^Kip1^ caused by AMPKα2 siRNA transfection. Our results further indicated that inhibition of Skp2 by siRNA abolished AMPKα2 knockdown-induced p27^Kip1^ reduction and therefore suppressed PASMCs proliferation. Taken together, these results suggest that Skp2/p27^Kip1^ pathway lays to the downstream of mTOR in mediating the effect of AMPKα2 on PASMCs proliferation. Encouragingly, our preliminary study in rat PAH model found that similar mechanisms were also existed *in vivo* (date not shown here).

PAH is a common clinical syndrome with high mortality and requires a longer-term management [1]. The present study demonstrates that knockdown of AMPKα2 stimulated PASMCs proliferation, suggesting that enhancing AMPKα2 activity might be a novel therapeutic strategy in the prevention and treatment of PAH. Metformin is an *in vitro* synthetic AMPK agonist which has been commonly used in clinic to treat type 2 diabetes with wide clinical experience and safety record [49]. We have recently found that activation of AMPKα2 by metformin suppresses PASMCs proliferation [50], together with our current findings, suggesting that metformin might be used in clinical treatment of PAH. Yet, this still needs to be tested in patients with PAH.

## 4. Materials and Methods

### 4.1. Cell Culture

Rat PASMCs were obtained from main pulmonary arteries from 4- to 5-week-old Sprague-Dawley rats using the method described by Wu *et al.* [17]. All animal procedures were approved and carried out in accordance with the Laboratory Animal Care Committee of Xi’an Jiaotong University. Briefly, rats were euthanized by CO_2_ overdose. Main pulmonary arteries were rapidly dissociated and cleaned under sterile conditions. Then, the adventitia and endothelium of pulmonary arteries were carefully scraped off and the remaining smooth muscle was minced (about 1 mm^2^) and placed into a culture flask. PASMCs were cultured in Dulbecco’s Modified Eagle Medium (DMEM)/High glucose (Gibco, Grand Isle, NY, USA) supplemented with 10% fetal bovine serum (FBS, Sijiqing, HangZhou, China) and 1% antibiotics (complete DMEM) in a humidified atmosphere of 5% CO_2_ and 95% air at 37 °C. Cells were fed every 2–3 days and subcultured by trypsinization using 0.25% trypsin (Invitrogen, Carlsbad, CA, USA) till reaching 80% confluence. All PASMCs used were from passages 4 and 6. The purity and identity of PASMC were determined by immunostaining with an antibody against α-smooth muscle actin (α-SMA, Sigma-Aldrich, St. Louis, MO, USA) as described previously [17].

### 4.2. siRNA Transfection

Transfection of PASMCs with siRNA (GenePharm, Shanghai, China) was performed using Lipofectamine™ 2000 transfection reagent (Invitrogen) according to the manufacture’s protocols. PASMCs were seeded into 6-well plates and cultured till reaching 30%–40% confluence. Cells were then transfected with 100 μM siRNA plus 5 μL of Lipofectamine for 6 h in serum-free DMEM and sequentially cultured in complete DMEM for 48 h in a 37 °C, 5% CO_2_ humidified incubator. The silencing efficiency was determined by immunoblotting.

### 4.3. BrdU Incorporation Assay

Cells proliferation was determine by BrdU incorporation assay according to the instructions of BrdU ELISA Kit (Maibio, Shanghai, China). Briefly, aliquots of 5 × 10^3^ cells were plated on 96-well plates and serum deprived (1% FBS in DMEM) overnight. After different treatments, cells were labeled by addition of BrdU for 2 h at 37 °C. After removing the labeling medium, cells were fixed and denatured by FixDenat solution and then incubated with anti-BrdU monoclonal antibody conjugated to peroxidase for 90 min at room temperature. Finally, substrate solution was added in each well and the absorbance at 370 nm was measured using a microplate reader (Bio-Rad, Richmond, CA, USA). Absorbance was normalized to initial reading to verify equal cell numbers at the start of assay.

### 4.4. Immunoblotting

The cultured PASMCs were washed twice with ice-cold phosphate buffered saline (PBS) and lysed on ice in RIPA lysis buffer containing 1 mM phenylmethanesulfonyl fluoride and proteinase inhibitors. The lysates were then centrifuged at 13,000 rpm for 15 min at 4 °C, and the supernatant was collected as sample protein. Equal amount of protein from each sample was loaded and resolved on sodium dodecyl sulfate-polyacrylamide gels and transferred to nitrocellulose membranes (Bio-Rad) using semidry transfer. The membranes were then blocked and probed with polyclonal or monoclonal primary antibodies against phosphor-mTOR, total-mTOR, Skp2, p27^Kip1^ (Cell Signaling Technology, Beverly, MA, USA; 1:1000 dilution), AMPKα1 and AMPKα2 (Proteintech Group, Chicago, IL, USA; 1:500 dilution) as well as glyceraldehyde-3-phosphate dehydrogenase (GAPDH, Sigma-Aldrich; 1:2000 dilution). After incubating with primary antibodies, the membranes were washed and incubated with horseradish peroxidase-conjugated secondary antibodies (Sigma-Aldrich; 1:5000 dilution). Immunoreactive bands were visualized by SuperSignal West Pico Chemiluminescent Substrate (Pierce Biotechnology, Rockford, IL, USA) and quantified using Quality One software (Bio-Rad). Sample loadings were normalized by immunoblotting with GAPDH or related total protein.

### 4.5. Statistics

Statistical analysis was carried out using the SPSS 13.0 software (SPSS Inc., Chicago, IL, USA). All data were expressed as mean ± standard deviation. Group comparisons were performed using one-way analysis of variance (ANOVA) followed by Tukey post hoc test. Statistical significant was determined at the level of *p* < 0.05.

## Abbreviations

AICAR	5-aminoimidazole-4-carboxamide ribonucleoside
AMPK	adenosine monophosphate-activated protein kinase
CDKs	cyclin-dependent kinases
mTOR	mammalian target of rapamycin
PAH	pulmonary arterial hypertension
PASMCs	pulmonary arterial smooth muscle cells
RNAi	RNA interference
Skp2	S-phase kinase-associated protein 2
siRNA	small interfering RNA
TSC	tuberous sclerosis complex
